# Femoral bone mineral density reference values by sex and ethnicity: Findings from the Qatar Biobank study

**DOI:** 10.1016/j.afos.2025.05.006

**Published:** 2025-06-11

**Authors:** Fawziya Al-Baker, Mujahed Shraim, Amal Al-Haidose, Atiyeh M. Abdallah

**Affiliations:** a Department of Biomedical Sciences, College of Health Sciences, QU-Health, Qatar University, Doha, Qatar; b Department of Public Health, College of Health Sciences, QU-Health, Qatar University, Doha, Qatar

**Keywords:** Bone mineral density, Osteoporosis, Reference values, Qatar Biobank, Population

## Abstract

**Objectives:**

Low bone mineral density (BMD) is a major global health concern due to fracture-related morbidity and mortality. BMD is currently assessed by dual-energy X-ray absorptiometry (DXA) against a US reference population. The aim of the study was to establish an ethnicity- and sex-specific reference for calculating BMD at different femoral sites including femoral trochanter, Ward's triangle and femoral neck in Qatar.

**Methods:**

This cross-sectional study analyzed BMD in 4727 (2277 females and 2450 males) healthy Qatari aged between 20 and 82 years participating in the Qatar Biobank (QBB) study. Standard T-scores provided by the densitometer (T_DXA_) were compared with ethnicity- and sex-specific T-scores for the Qatari population (T_QA_) calculated using data from the 20-29-year age group for different femoral sites as the reference. The concordance between T_DXA_ and T_QA_ was analyzed using kappa statistics.

**Results:**

Males consistently exhibited higher BMD values for the femoral trochanter, Ward's triangle, and the femoral neck across all age groups compared with females. Using T_QA_ rather than T_DXA_ as a reference at any site classified fewer individuals as having osteoporosis and osteopenia, especially for females. Agreement between T_DXA_ and T_QA_ was higher in males than in females.

**Conclusions:**

These findings underscore the need for local ethnicity- and sex-specific BMD reference values. The use of T_QA_ significantly reduced overdiagnosis of osteopenia and osteoporosis compared with T_DXA_, thereby decreasing overtreatment and impacting participant recruitment decisions into clinical studies.

## 1 Introduction

Osteoporosis is a systemic skeletal disease characterized by low bone mineral density (BMD) and disruption of the bone microarchitecture through trabecular thinning, decreased cortical thickness, and a deterioration of bone tissues. These processes ultimately compromise bone strength and increase the risk of fracture [1]. Osteoporosis is underpinned by imbalanced bone homeostasis caused by decreased bone formation or increased bone resorption [2]. It is a global and increasing health problem, with the International Osteoporosis Foundation (IOF) estimating that osteoporosis causes over 8.9 million fractures annually worldwide and that one-third of females and one-fifth of males over 50 years will experience an osteoporotic fracture in their lifetime [3].

Therefore, the early identification and detection of bone pathology are critical for the prevention of bone loss and the effective treatment of conditions like osteoporosis. Pathological changes in bone structure and density are assessed using a variety of clinical and laboratory techniques that allow a diagnosis of osteoporosis, including single or dual photon absorptiometry, quantitative compound tomography, neutron and proton activation analysis, and quantitative magnetic resonance [4]. Of these, dual X-ray absorptiometry (DXA), which is accurate, precise, and has low radiation exposure, is the gold standard for measuring peripheral and central BMD and for diagnosing and monitoring osteoporosis [[5], [6], [7]]. Using DXA, BMD is typically measured at specific sites, including the lumbar vertebrae, femoral neck, and hip, although this can lead to variability in peak BMD values according to the non-uniform measurement locations specified in the WHO's diagnostic criteria for osteoporosis [2].

An individual's BMD is commonly expressed as a T-score [8], calculated by subtracting the mean BMD of the reference population from the patient's BMD and dividing this difference by the standard deviation (SD) of the reference population [9]. In many countries, T-score levels defined by the WHO serves as both diagnostic and intervention thresholds. The WHO reference standard is based on females in the US participating in the third National Health and Nutrition Evaluation Survey (NHANES III), regardless of race or sex/gender, and the reference is based on the assumption that peak femoral BMD is typically reached at around 25 years of age [10]. The International Society for Clinical Densitometry (ISCD) report that fractures occur at comparable BMDs in males and females, so they suggest using this uniform White, non-ethnicity-adjusted female reference for both sexes [10,11]. Most research on osteoporosis has focused on females due to their greater risk of osteoporosis and subsequent fractures [12]. However, given that the genetic, nutritional, cultural, and lifestyle factors that affect bone health vary significantly between populations, using a young White reference in an ethnically quite different population such as that seen in Qatar could lead to overdiagnosis or underdiagnosis of osteoporosis.

Therefore, the objectives of this study were to: 1) describe the BMD distribution at various femoral sites in males and females; 2) identify sex- and age-specific reference values for calculating T-scores for the Qatari population; and 3) analyze the prevalence of osteopenia and osteoporosis in the Qatari population based on the newly established T-scores.

## 2 Methods

### 2.1 Ethical approval

The Qatar Biobank Institutional Review Board (E−2021-QF-QBB-RES-ACC-00050-0172) and 10.13039/501100004252Qatar University Institutional Review Board (1648-E/22) granted ethical approval for this study. All participants provided informed consent to share their data for research governed by an information security management system implemented by QBB to guarantee the confidentiality and integrity of all participant information [13].

### 2.2 Study population

Data from Qatar Biobank (QBB) was used for this cross-sectional study. The inclusion criteria were healthy Qatari, without a history of major chronic conditions, aged between 20 and 82 years, the wide age range chosen to track BMD progression across different life stages within the Qatari population. Participants with chronic illnesses known to impact BMD such as diabetes mellitus, all cancers, thyroid diseases, inflammatory bowel diseases such as Crohn's disease and ulcerative colitis, prior fractures, autoimmune diseases, arthritis, and gastrointestinal surgeries were excluded. Participants underwent comprehensive health assessments for a wide range of factors including lifestyle and sociodemographic characteristics [13,14].

### 2.3 BMD measurements

BMD was assessed by certified professionals at the QBB using the Lunar Prodigy (GE Healthcare, Madison, WI, USA) system with Encore Software. BMD measurements were taken from various femoral sites prone to fractures including the femoral trochanter, Ward's triangle, and the femoral neck, which are crucial for diagnosing and evaluating fracture risk. DXA measurements from male and female participants, segmented into specific age categories, were analyzed to determine T-scores. Osteoporosis was diagnosed based on WHO criteria, where T-scores less than or equal to −1 were normal, between −1 and −2.5 indicative of low bone mass (osteopenia), and −2.5 or lower as osteoporosis [1,10,15]. In this study, T-scores were compared based on two reference values: (i) the standard reference, denoted (T_DXA_), was based on the established standard from the NHANES III data from young White female adults, which serves as a benchmark for international comparison as per the manufacturer's recommendation and current practice within the QBB; and (ii) the local Qatar reference, denoted (T_QA_), derived from the mean and standard deviation (SD) of BMD from males and females separately in the 20–29 years age group to calculate sex-specific T-scores. This age group was selected as the reference population in accordance with WHO and ISCD guidelines, which recommend defining T-scores based on BMD in young adults. This standardization allowed for consistency across studies and facilitated comparison with internationally accepted thresholds.

### 2.4 Data analysis

For comparison with NHANES, we combined BMD data into standard age groups similar to other studies (20–29, 30–39, 40–49, 50–59 and ≥ 60) for the three femoral sites (femoral neck, trochanter, and Ward's triangle), which show distinct BMD patterns according to sex. Participants ≥ 60 years were grouped separately.

Data were analyzed using Stata/SE v18 software. Data were preprocessed and cleaned with management of missing values, comprehensive review and removal of outliers, and assessment of skew and normality to ensure the quality of the data prior to further analysis. Then, descriptive statistics were computed for normalized variables, expressing data as means and standard deviations (SD) for 4727 participants stratified by sex and age group. Using age group, femoral site, sex, and BMD values, T-scores were calculated and classified according to WHO criteria into normal, osteopenia, or osteoporosis. Statistical agreement between T_DXA_ and T_QA_ was analyzed using kappa statistics with 95% confidence intervals (CI).

## 3 Results

### 3.1 Demographic characteristics of the study population

BMD data from 4727 participants were analyzed, and their demographic characteristics are summarized in Table 1. The cohort included 2277 females (48.2%) and 2450 males (51.8%) with a mean (SD) age of 34.6 (10.2) years for females and 35.2 (10.0) years for males.

**Table 1:** Baseline characteristics of study participants (N = 4727). Values are presented as mean (SD).

Variable	Female N = 2277 (48.2%)	Male N = 2450 (51.8%)	P-value
Age, yrs	34.63 (10.17)	35.16 (9.93)	0.05
BMI, kg/m^2^	28.30 (6.27)	28.15 (5.36)	0.88
Height, cm	158.40 (5.79)	173.01 (6.48)	<0.05
Weight, kg	70.97 (16.13)	84.41 (17.52)	<0.05
Ward's triangle BMD, g/cm^2^	0.804 (0.146)	0.885 (0.174)	<0.05
Femoral trochanter BMD, g/cm^2^	0.767 (0.118)	0.877 (0.135)	<0.05
Femoral neck BMD, g/cm^2^	0.942 (0.124)	1.035 (0.152)	<0.05

### 3.2 BMD in Qatar

Males consistently exhibited higher BMD values across all age groups compared with females (Table 2), with the highest BMD values observed in males aged 20–29 at the femoral neck (BMD 1.072 (0.163) g/cm^2^). The highest BMD in females was within the 40–49 age group at the femoral neck (0.959 (0.126) g/cm^2^). BMD measured at the femoral trochanter was higher in males than in females, peaking at 0.914 (0.127) g/cm^2^ in the 50–59 age group and in females at 0.806 (0.119) g/cm^2^ in the 40–49 age group. The highest mean BMD at Ward's triangle was in the 20–29 age group for both sexes, with a BMD of 0.952 (0.182) g/cm^2^ in males and 0.835 (0.142) g/cm^2^ in females.

**Table 2:** BMD measurements across three different femoral sites in males and females. Values are presented as mean (SD). BMD values are in g/cm^2^.

Age group	Count	Femoral trochanter	Ward's triangle	Femoral neck
**Female**				
20–29	839	0.744 (0.117)	0.835 (0.142)	0.936 (0.125)
30–39	739	0.764 (0.113)	0.800 (0.137)	0.948 (0.121)
40–49	475	0.806 (0.119)	0.795 (0.153)	0.959 (0.126)
50–59	193	0.785 (0.112)	0.733 (0.133)	0.915 (0.120)
≥60	31	0.748 (0.100)	0.644 (0.116)	0.863 (0.114)
**Male**				
20–29	845	0.878 (0.144)	0.952 (0.182)	1.072 (0.163)
30–39	873	0.867 (0.127)	0.875 (0.158)	1.030 (0.141)
40–49	525	0.880 (0.132)	0.830 (0.154)	1.007 (0.143)
50–59	157	0.914 (0.127)	0.815 (0.141)	1.006 (0.133)
≥60	50	0.861 (0.155)	0.705 (0.158)	0.910 (0.156)

### 3.3 T-score calculations for the Qatari population

**Fig. 1:**
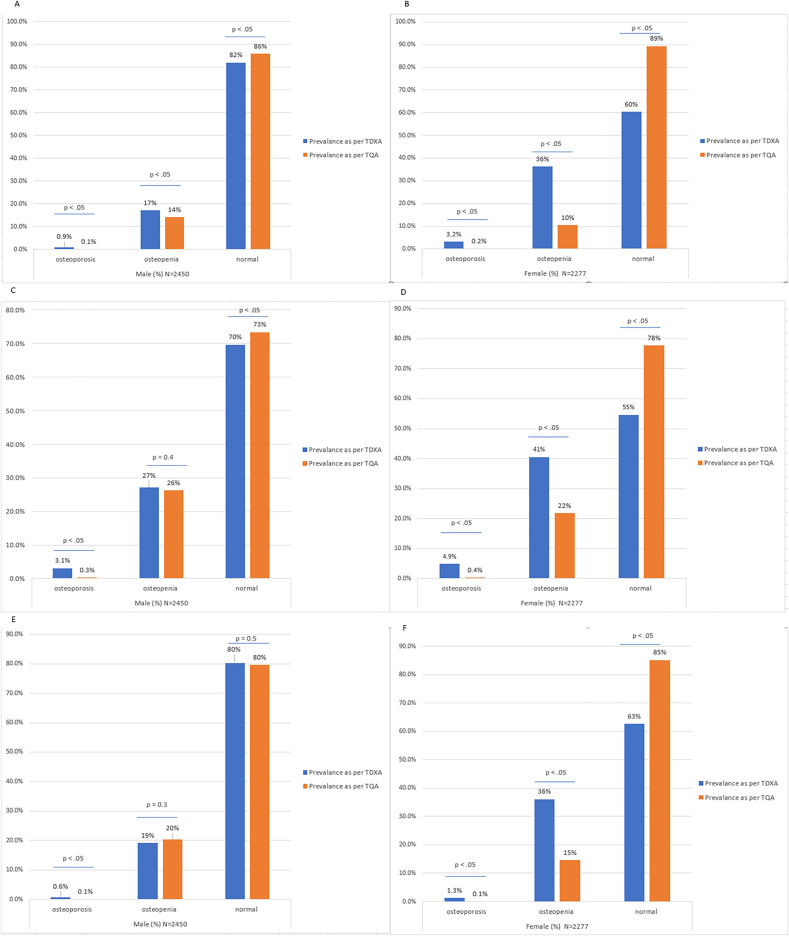
Comparative prevalence of osteopenia and osteoporosis measured at the femoral trochanter (A, males; B females), Ward's triangle (C, males; D females), and femoral neck (E, males; F females) using different standards. T_DXA_, T-scores provided by the densitometer; T_QA_, T-scores for the Qatari population.

The local Qatari population aged 20–29 years was used as a reference group to calculate ethnicity- and sex-specific T-scores, denoted "T_QA_", which were compared with T-scores provided by the densitometer (T_DXA_). The number of individuals with a low femoral BMD calculated using T_QA_ was lower than that determined by T_DXA_. At the femoral trochanter, more males and a significantly larger proportion of females were identified as having a normal bone density using T_QA_ than with T_DXA_. Furthermore, 0.9% of males were defined as having osteoporosis using T_DXA_ compared to only 0.1% using T_QA_ (P < 0.05; Fig. 1A). Similarly, 3.2% of females had osteoporosis at the femoral trochanter using T_DXA_ compared with only 0.2% using T_QA_ (P < 0.05; Fig. 1B). At Ward's triangle, 3.10% of males had osteoporosis using T_DXA_ compared with only 0.30% using T_QA_ (P < 0.05; Fig. 1C). Similarly, 4.90% of females had osteoporosis at the femoral trochanter using T_DXA_ compared with 0.40% using T_QA_ (P < 0.05; Fig. 1D). At the femoral neck, T_DXA_ defined 80.3% of males as having a normal bone density compared with 79.6% by T_QA_ (P = 0.5; Fig. 1E). By contrast, 62.7% of females had a normal BMD by T_DXA_ compared with 85.2% by T_QA_ (P < 0.05; Fig. 1F). The prevalence of diagnoses for the entire study population divided into age groups is presented in Table 3, Table 4 for females and males, respectively.

**Table 3:** Prevalence of osteopenia and osteoporosis in the female Qatari population using both the T_QA_ and T_DXA_ according to different age groups.

Age Group	Condition	T_QA_			T_DXA_		
		Femoral trochanter N (%)	Ward's triangle N (%)	Femoral neck N (%)	Femoral trochanter N (%)	Ward's triangle N (%)	Femoral neck N (%)
20–29	Normal	711 (84.7)	707 (84.3)	709 (84.5)	444 (52.9)	538 (64.1)	510 (60.8)
	Osteopenia	123 (14.7)	130 (15.5)	128 (15.3)	354 (42.2)	279 (33.3)	312 (37.2)
	Osteoporosis	5 (0.6)	2 (0.2)	2 (0.2)	41 (4.9)	22 (2.6)	17 (2.0)
30–39	Normal	660 (89.3)	581 (78.6)	639 (86.5)	436 (59.0)	388 (52.5)	478 (64.7)
	Osteopenia	79 (10.7)	158 (21.4)	100 (13.5)	282 (38.2)	322 (43.6)	259 (35.0)
	Osteoporosis	0 (0.0)	0 (0)	0 (0)	21 (2.8)	29 (3.9)	2 (0.3)
40–49	Normal	455 (95.8)	356 (74.9)	425 (89.5)	347 (73.1)	241 (50.7)	318 (66.9)
	Osteopenia	20 (4.2)	119 (25.1)	50 (10.5)	123 (25.9)	211 (44.4)	154 (32.4)
	Osteoporosis	0 (0.0)	0 (0)	0 (0)	5 (1.1)	23 (4.8)	3 (0.6)
50–59	Normal	181 (93.8)	117 (60.6)	149 (77.2)	132 (68.4)	74 (38.3)	111 (57.5)
	Osteopenia	12 (6.2)	71 (36.8)	43 (22.3)	56 (29.0)	93 (48.2)	76 (39.4)
	Osteoporosis	0 (0.0)	5 (2.6)	1 (0.5)	5 (2.6)	26 (13.5)	6 (3.1)
≥60	Normal	28 (90.3)	10 (32.3)	19 (61.3)	16 (51.6)	3 (9.7)	10 (32.3)
	Osteopenia	3 (9.7)	19 (61.3)	12 (38.7)	14 (45.2)	17 (54.8)	20 (64.5)
	Osteoporosis	0 (0.0)	2 (6.5)	0 (0)	1 (3.2)	11 (35.5)	1 (3.2)

**Table 4:** Prevalence of osteopenia and osteoporosis in the male Qatari population using both the T_QA_ and T_DXA_ according to different age groups.

Age Group	Condition	T_QA_			T_DXA_		
		Femoral trochanter N (%)	Ward's triangle N (%)	Femoral neck N (%)	Femoral trochanter N (%)	Ward's triangle N (%)	Femoral neck N (%)
20–29	Normal	719 (85.1)	713 (84.4)	709 (83.9)	690 (81.7)	692 (81.9)	716 (84.7)
	Osteopenia	125 (14.8)	131 (15.5)	135 (16.0)	148 (17.5)	146 (17.3)	128 (15.1)
	Osteoporosis	1 (0.1)	1 (0.1)	1 (0.1)	7 (0.8)	7 (0.8)	1 (0.1)
30–39	Normal	741 (84.9)	659 (75.5)	703 (80.5)	706 (80.9)	620 (71.0)	708 (81.1)
	Osteopenia	132 (15.1)	214 (24.5)	170 (19.5)	157 (18.0)	227 (26.0)	160 (18.3)
	Osteoporosis	0 (0)	0 (0)	0 (0)	10 (1.1)	26 (3.0)	5 (0.6)
40–49	Normal	460 (87.6)	318 (60.6)	395 (75.2)	435 (82.9)	294 (56.0)	396 (75.4)
	Osteopenia	65 (12.4)	207 (39.4)	130 (24.8)	87 (16.6)	207 (39.4)	125 (23.8)
	Osteoporosis	0 (0)	0 (0)	0 (0)	3 (0.6)	24 (4.6)	4 (0.8)
50–59	Normal	144 (91.7)	95 (60.5)	122 (77.7)	140 (89.2)	90 (57.3)	124 (79.0)
	Osteopenia	13 (8.3)	61 (38.9)	35 (22.3)	17 (10.8)	61 (38.9)	33 (21.0)
	Osteoporosis	0 (0)	1 (0.6)	0 (0)	0 (0.0)	6 (3.8)	0 (0)
≥60	Normal	38 (76.0)	13 (26.0)	22 (44.0)	37 (74.0)	12 (24.0)	24 (48.0)
	Osteopenia	11 (22.0)	31 (62.0)	27 (54.0)	11 (22.0)	25 (50.0)	22 (44.0)
	Osteoporosis	1 (2.0)	6 (12.0)	1 (2.0)	2 (4.0)	13 (26.0)	4 (8.0)

### 3.4 Agreement of diagnosis

Kappa (κω) statistics with 95% CI were used to evaluate the diagnostic agreement between T_DXA_ and T_QA_ at the three femoral sites (Table 5). There were varying levels of agreement across different age groups and sexes according to standard definitions [16]. In females, there was slight to moderate agreement across all femoral sites. For individuals aged 20–29, the κω was 0.324 for the femoral trochanter, 0.431 at the femoral neck, and 0.478 at Ward's triangle, and the agreement was similar for the other age groups. The lowest κω of 0.192 (95% CI: 0.000–0.446) was in the ≥ 60 age group at the femoral trochanter, and the highest was at the femoral neck for the 50–59 age group, with a κω of 0.550 (95% CI: 0.446–0.656).

**Table 5:** Diagnostic agreement for osteoporosis at different femoral sites for T_DXA_ and T_QA_.

				Femoral trochanter T_QA_				Femoral neck T_QA_				Ward's triangle T_QA_			
	Gender	Age group		Normal	Osteopenia	Osteoporosis	Kappa (BC 95% CI)	Normal	Osteopenia	Osteoporosis	Kappa (BC 95% CI)	Normal	Osteopenia	Osteoporosis	Kappa (BC 95% CI)
T_DXA_	Female	20–29	Normal	444	0	0	0.324 (0.279–0.371)	510	0	0	0.431 (0.379–0.485)	538	0	0	0.478 (0.425–0.530)
			Osteopenia	267	87	0		199	113	0		169	110	0	
			Osteoporosis	0	36	5		0	15	2		0	20	2	
		30–39	Normal	436	0	0	0.276 (0.229–0.327)	478	0	0	0.442 (0.382–0.503)	388	0	0	0.428 (0.377–0.479)
			Osteopenia	224	58	0		161	98	0		193	129	0	
			Osteoporosis	0	21	0		0	2	0		0	29	0	
		40–49	Normal	347	0	0	0.205 (0.133–0.283)	318	0	0	0.378 (0.301–0.459)	241	0	0	0.467 (0.406–0.525)
			Osteopenia	108	15	0		107	47	0		115	96	0	
			Osteoporosis	0	5	0		0	3	0		0	23	0	
		50–59	Normal	132	0	0	0.233 (0.132–0.350)	111	0	0	0.550 (0.446–0.656)	74	0	0	0.511 (0.428–0.599)
			Osteopenia	49	7	0		38	38	0		43	50	0	
			Osteoporosis	0	5	0		0	5	1		0	21	5	
		≥60	Normal	16	0	0	0.192 (0.000–0.446)	10	0	0	0.436 (0.236–0.701)	3	0	0	0.293 (0.104–0.526)
			Osteopenia	12	2	0		9	11	0		7	10	0	
			Osteoporosis	0	1	0		0	1	0		0	9	2	
	Male	20–29	Normal	690	0	0	0.856 (0.810–0.897)	705	11	0	0.933 (0.898–0.963)	692	0	0	0.890 (0.847–0.925)
			Osteopenia	29	119	0		4	124	0		21	125	0	
			Osteoporosis	0	6	1		0	0	1		0	6	1	
		30–39	Normal	706	0	0	0.826 (0.778–0.868)	692	16	0	0.884 (0.842–0.920)	620	0	0	0.824 (0.787–0.860)
			Osteopenia	35	122	0		11	149	0		39	188	0	
			Osteoporosis	0	10	0		0	5	0		0	26	0	
		40–49	Normal	435	0	0	0.794 (0.717–0.862)	385	11	0	0.874 (0.824–0.919)	294	0	0	0.828 (0.785–0.868)
			Osteopenia	25	62	0		10	115	0		24	183	0	
			Osteoporosis	0	3	0		0	4	0		0	24	0	
		50–59	Normal	140	0	0	0.853 (0.712–0.994)	120	4	0	0.887 (0.799–0.976)	90	0	0	0.880 (0.808–0.940)
			Osteopenia	4	13	0		2	31	0		5	56	0	
			Osteoporosis	0	0	0		0	0	0		0	5	1	
		≥60	Normal	37	0	0	0.908 (0.749–1.000)	22	2	0	0.832 (0.693–0.963)	12	0	0	0.769 (0.609–0.906)
			Osteopenia	1	10	0		0	22	0		1	24	0	
			Osteoporosis	0	1	1		0	3	1		0	7	6	

By contrast, males exhibited higher kappa values and thus better agreement between the two references for all femoral sites, ranging from substantial to several instances of almost perfect agreement. For instance, the lowest κω of 0.769 (95% CI: 0.609–0.906) was observed in the ≥ 60 age group at Ward's triangle, and the highest κω of 0.933 (95% CI: 0.898–0.963) was in the 20–29 age group at the femoral neck. Therefore, stronger agreement between T_DXA_ and T_QA_ was observed in males, suggesting closer alignment between the classification compared to females.

## 4 Discussion

Bone mineral density testing is an essential component of the diagnosis and treatment of bone disorders, especially osteoporosis. The standardized reference values used worldwide for T-score calculation are generated from a young healthy White population aged 20–29 years [1]. These values cannot adequately reflect bone health state across diverse ethnic and geographic populations [17]. Numerus studies have suggested that BMD varies according to geographical area, age, ethnicity, and sex/gender groups [18]. For instance, T-scores were found to be lower in Gulf countries including Oman, United Arab Emirates (UAE), and Qatar than the reference White female population used for DXA measurements, with differences observed even between these countries [19,20]. Recognizing this gap, many countries have set up their own reference standards based on their populations and that reflect their citizens' distinct bone health profiles [[21], [22], [23]]. By taking a similar strategy, Qatar could enhance the precision of bone health evaluations, resulting in more targeted healthcare treatments and reducing the risk of over- or underdiagnosis of osteoporosis and osteopenia.

We detected a significant discrepancy in BMD values obtained from the T_DXA_ reference and our estimated T_QA_ values, with further differences according to demographics. For instance, the mean T_QA_ BMD at the femoral neck was 0.936 (0.125) for females and 1.072 (0.163) for males, considerably lower than the T_DXA_-derived reference values of 1.038 (0.139) for females but the same [1.070 (0.130)] in males [24]. This finding is consistent with similar studies performed in Indian, Lebanese, and other Asian populations, further emphasizing the need for local BMD reference values [17,21,25]. These differences also had clinical consequences. Using the international T_DXA_ reference resulted in a significantly higher prevalence of osteoporosis, particularly among females, compared to the local T_QA_ reference. This suggests that the standard reference may not be appropriately calibrated to the Qatari population and could lead to overdiagnosis. This was also observed in a Vietnamese study, where the application of NHANES reference values resulted in 35% overdiagnosis of osteoporosis and osteopenia compared with using a local reference [8]. Overdiagnosis could result in unnecessary management and patient anxiety as well as erroneously increasing the prevalence of osteoporosis in public health data [12].

Males in our cohort generally had higher mean BMD values than females, and our T_QA_-referenced BMD values revealed sex-specific patterns that are important for understanding bone health throughout life stages (Table 3, Table 4). In females, we observed that BMD values increased during early adulthood, peaking in the 40–49 age group at the femoral neck and trochanter, and in the 30–39 age group at Ward's triangle, before declining in older age groups. Indeed, this disparity was emphasized in the 50–59 age group, with females experiencing a more dramatic decrease in BMD together with a higher prevalence of osteopenia. In individuals aged ≥ 60 years, we observed high rates of normal BMD, although this result was influenced by the small sample size within this subgroup. This small cohort of individuals aged ≥ 60 years with relatively good BMD may be also explained by changes in lifestyles in Qatar, who are not so exposed to unhealthy behaviors such as smoking, caffeine, and carbonated beverage consumption, which are endemic in young adults in Qatar [28,29]. Our observations are also consistent with studies showing that males typically have higher peak bone mass and experience bone loss at a slower rate as they age compared with females, although BMD in males peaked later, at 50–59 years, at the femoral trochanter [30]. Sex-related decreases in bone density are well recognized and are largely attributable to hormonal changes, particularly those occurring before and after menopause, which place women at greater risk of reduced BMD and bone fractures [20,26,27,31].

The observed sex-related differences became even more obvious when comparing bone health between the two references across the entire study cohort. For example, when comparing the values derived from the femoral trochanter, there was a significant 29% difference in normal BMD in females versus 3.8% in males for the two sets of measurements, equating to a 26% and 3% decrease in osteopenia prevalence when using T_QA_ for females and males, respectively, and a 3% and 0.8% decrease in osteoporosis prevalence for females and males, respectively. This trend towards overestimation was also observed for Ward's triangle and femoral neck measurements. Over-diagnosis is a widespread and well-recognized problem in non-Western cultures when standard reference values are utilized [21,25,32]. By contrast, a study from Ireland reported that the standard NHANES III reference values are suitable for other populations, arguing that this standard reflects the pattern seen in a healthy population [24]. Nevertheless, this approach assumes that the standard reference is sufficiently similar to the characteristics of the local population.

Assessment of agreement between values derived using the two references revealed fair to moderate agreement for females, suggesting the potential for overestimation of the diagnosis, and even greater agreement for males. Nevertheless, by adopting the T_QA_ standard, Qatar can more accurately reflect its population's bone health characteristics, increasing the accuracy of diagnosis and the appropriateness of subsequent treatments.

This study has some limitations. This study did not take into consideration other confounding variables that could affect BMD, such as BMI, lifestyle, vitamin and drug use, dietary habits, and genetic predisposition. In particular, given that the study population exhibited a relatively high BMI and considering the known correlation between BMI and BMD, future studies should explore this relationship, including across age groups. Another limitation was the relatively small number of our participants over the age of 60, which reduces the generalizability of our findings to older populations, who are at particularly high risk for osteoporosis. Therefore, future studies should include a broader representation of older individuals and a wider range of variables to gain more accurate insights into bone health in the Qatari population. Future studies should also incorporate additional clinical indicators such as fracture incidence to validate the diagnostic performance and clinical applicability of the T_QA_ reference values. Finally, in our analysis we did not specifically assess the age of peak BMD within the Qatari population; therefore, our use of the 20–29 age group as the reference, while consistent with WHO/ISCD recommendations, assumes that this age range represents peak BMD locally, which may not be fully validated.

## 5 Conclusions

This study highlights the importance of developing localized BMD reference values to accurately assess the risk of osteoporosis and bone health in the Qatari population. Using a large cohort of healthy participants in the QBB, we compared BMD values derived using the standard reference with a new Qatari reference derived from the same age category. Implementation of the T_QA_ reference values in Qatar could improve the accuracy of diagnosis and hence the management of patients with osteopenia and osteoporosis in Qatar. However, more studies are needed to analyze the new reference values in patients with osteopenia, osteoporosis and bone fractions.

## CRediT author statement

**Fawziya Al-Baker:** Statistical analysis, Writing – original draft, Writing – review & editing. **Mujahed Shraim:** Methodology, Statistical analysis, Writing – review & editing. **Amal Al-Haidose:** Supervision, Funding acquisition, Writing – original draft, Writing – review & editing. **Atiyeh M. Abdallah:** Conceptualization, Methodology, Supervision, Funding acquisition, Writing – review & editing.

## Conflicts of interest

The authors declare no competing interests.
